# Sensitive Skin in Thais: Prevalence, Clinical Characteristics, and Diagnostic Cutoff Scores

**DOI:** 10.1111/jocd.70181

**Published:** 2025-04-11

**Authors:** Pichanee Chaweekulrat, Silada Kanokrungsee, Noldtawat Viriyaskultorn, Suthasanee Prasertsook, Surachanee Likittanasombat, Waranya Boonchai

**Affiliations:** ^1^ Department of Dermatology Faculty of Medicine Siriraj Hospital, Mahidol University Bangkok Thailand

**Keywords:** burden of sensitive skin, questionnaire, sensitive scale, sensitive skin syndrome

## Abstract

**Background:**

Sensitive skin is a dermatologic condition with variable prevalence. Universally established cutoff scores for the sensitive scale (SS) and burden of sensitive skin (BoSS) questionnaires are lacking in general populations.

**Aims:**

To determine the prevalence of and the associated risk factors for sensitive skin among Thais and to establish cutoff values for diagnosing mild, moderate, and severe cases of sensitive skin.

**Patients/Methods:**

A cross‐sectional study involving 621 participants aged ≥ 18 years was conducted using an online questionnaire disseminated via various social platforms. Participants completed the SS‐14, SS‐10, and BoSS questionnaires to assess sensitive skin severity. Cutoff scores for these instruments were determined.

**Results:**

Sensitive skin was reported by 86.9% of participants, with 57.5% indicating moderate to severe sensitive skin. Significant risk factors for sensitive skin included female sex, underlying dermatologic conditions, history of atopic dermatitis, and dry skin type. The following cutoff points for diagnosing mildly, moderately, and severely sensitive skin were established for each questionnaire: SS‐14 (6/16/25), SS‐10 (5/13/22), and BoSS (13/19/24), respectively. The SS‐10 questionnaire demonstrated greater diagnostic accuracy than the BoSS questionnaire.

**Conclusions:**

This pioneering study elucidated the prevalence of and risk factors for sensitive skin in Thais. The established cutoff values will facilitate sensitive skin diagnosis and guide patient management strategies.

## 1 Introduction

Sensitive skin syndrome is characterized by unpleasant sensations, such as pruritus, burning, pain, and tingling, in response to typically innocuous stimuli [1]. This condition primarily affects the face but can manifest elsewhere, often without visible clinical signs, except for occasional erythema. Although the etiology of the syndrome remains unclear, research suggests associations with skin barrier alterations and neurosensory hyperactivity [2]. Precipitating factors for sensitive skin syndrome include environmental stimuli (ultraviolet radiation, wind, and temperature extremes), chemical agents (such as pollutants and cosmetics), and physiological factors (stress and the menstrual cycle) [1, 3]. The syndrome's prevalence exhibits significant variability across populations and geographic regions, with self‐reported rates ranging from 23% to 92% [4].

Given the subjective nature of sensitive skin syndrome, several assessment tools have been developed to quantify and monitor clinical severity. Notable among these are the sensitive scale (SS) questionnaire and the burden of sensitive skin (BoSS) questionnaire [5, 6]. The SS, initially a 14‐item instrument (SS‐14), has been refined to a 10‐item version (SS‐10) that focuses exclusively on sensitive symptoms and erythema, omitting visible signs [5]. The SS‐10 has demonstrated superior reliability compared with the SS‐14 and is preferred in clinical settings. However, universally accepted diagnostic cutoff values for the SS questionnaires remain elusive, with previous studies reporting varying thresholds for identifying sensitive skin [7, 8]. This variability underscores the need for further research to establish standardized diagnostic criteria for sensitive skin syndrome.

Sensitive skin significantly impacts patients' quality of life, akin to other dermatological conditions [2, 9]. The BoSS questionnaire was developed to evaluate severity of sensitive skin by assessing its impact on a patient's quality of life across three dimensions: self‐care, daily life, and self‐appearance [6]. Previous studies have indicated that the BoSS questionnaire not only satisfactorily measures quality of life but also may be a potential diagnostic tool [6, 8, 10].

Given the variation in cutoff scores for diagnosing sensitive skin, determining population‐specific cutoffs is crucial to facilitate diagnosis. This study examined the prevalence of and risk factors for sensitive skin within the Thai population. Additionally, we sought to establish cutoff values for the SS and BoSS questionnaires to enhance the diagnosis of sensitive skin in this population.

## 2 Materials and Methods

This questionnaire‐based, cross‐sectional study was conducted at the Department of Dermatology, Faculty of Medicine Siriraj Hospital, Mahidol University, Bangkok, Thailand. A total of 621 individuals participated in the study. All were 18 years or older and could read and respond to an online questionnaire in Thai. The questionnaire was distributed to the general population via a Google Form and was publicized across various online social platforms, including the LINE mobile messenger application and Facebook. Access to the questionnaire was also facilitated through invitation links.

The initial page of the questionnaire provided study information and informed consent. Participants were required to provide informed consent before completing the questionnaire. The study protocol was authorized by the Siriraj Institutional Review Board (approval number Si‐1021/2021).

### 2.1 Questionnaire

The questionnaire consisted of two sections. The first section focused on demographic information, including age, sex, and body mass index. It also covered underlying dermatologic conditions at the sensitive skin site, personal and family history of atopic conditions, physical activities, smoking status, skin type, Fitzpatrick skin type, menstruation status, and aggravating factors of sensitive skin.

The second section addressed characteristics and severity of sensitive skin. The definition of sensitive skin proposed by the International Forum for the Study of Itch was provided at the beginning of this section [1]. Participants were then asked, “Based on the definition, do you consider yourself to have sensitive skin?” They assessed their skin as “not sensitive at all,” “mildly sensitive,” “moderately sensitive,” or “severely sensitive.” Subsequently, participants completed the Thai versions of the SS‐14 and SS‐10 questionnaires to evaluate severity of their sensitive skin. Additionally, the BoSS questionnaire was administered to assess the impact of sensitive skin on quality of life. The Thai versions of the SS‐14, SS‐10, and BoSS questionnaires used in this study have been previously validated [11].

### 2.2 Statistical Analysis

Quantitative variables are described using means and standard deviations or medians and interquartile range (IQR), whereas qualitative variables are presented as percentages. Participants who reported having mildly, moderately, or severely sensitive skin were categorized into the “sensitive skin group” and the remaining participants were classified into the “nonsensitive skin group.”

The Kruskal–Wallis test was used to measure the difference in severity scores between the sensitive and nonsensitive skin groups and between the three sensitive‐severity subgroups. Pearson's correlation coefficient was used for correlation analysis to evaluate the associations of potential risk factors with sensitive skin. Risk factors with a significance level below a predefined cutoff value (*p* = 0.20) were included in a multivariate logistic regression model to assess the adjusted odds ratios (aORs).

Receiver operating characteristic (ROC) curves were generated to establish cutoff scores for the SS‐14, SS‐10, and BoSS questionnaires for diagnosing mildly, moderately, and severely sensitive skin, with subsequent calculations of sensitivity and specificity. An area under the curve (AUC) of 0.7–0.8 was considered acceptable, 0.8–0.9 was deemed excellent, while a value greater than 0.9 was regarded as outstanding [7]. The DeLong test was used to assess the significance of differences in the AUC values of ROC curves among these questionnaires. Statistical analyses were performed using IBM SPSS Statistics, version 22 (IBM Corp, Armonk, NY, USA). A two‐sided *p* value ≤ 0.05 was considered statistical significance.

## 3 Results

Among the 621 participants, the majority were female (81.6%). The mean age was 38.5 ± 12.0 years, and most participants lived in central Thailand (74.7%). Of all participants, 540 (86.9%) reported experiencing some degree of sensitive skin, with 183 (29.4%), 250 (40.2%), and 107 (17.2%) classifying themselves as having mildly, moderately, and severely sensitive skin, respectively. The facial area was the most affected region (72.3%), followed by extremities (61.7%), trunk (33.8%), head and neck (30.7%), hands or feet (28.8%), and genital area (7.5%). The demographic data are shown in Table 1.

**TABLE 1:** Demographic data and factors associated with sensitive skin.

	Total (*n* = 621)	Sensitive skin (*n* = 540)	Nonsensitive skin (*n* = 81)	Crude OR (95% CI)	*p*	Adjusted OR (95% CI)	*p*
Sex, *n* (%)							
Female	508 (81.8)	461 (85.4)	47 (58.0)	4.16 (2.52–6.86)	< 0.001*	3.98 (1.94–8.16)	< 0.001*
Male	113 (18.2)	79 (14.6)	34 (42.0)	1.00 [ref]	1.00	—	—
Age, y, mean ± SD	38.50 ± 12.00	39.78 ± 11.63	33.57 ± 13.05	1.00 (0.99–1.02)	0.792	—	—
Age group, y, *n* (%)							
18–39	337 (54.3)	292 (54.1)	45 (55.6)	0.94 (0.59–1.51)	0.803		
40–59	231 (37.2)	203 (37.6)	28 (34.6)	1.14 (0.70–1.86)	0.599		
> 60	53 (8.5)	45 (8.3)	8 (9.9)	0.83 (0.38–1.83)	0.643		
BMI (kg/m^2^), median (IQR)	22.7 (20.2, 25.7)	22.7 (20.1, 25.7)	22.9 (20.4, 25.1)	0.99 (0.94–1.04)	0.649	—	—
Underlying dermatologic disease, *n* (%)	333 (53.6)	326 (60.4)	7 (8.6)	16.10 (7.28–35.63)	< 0.001*	16.15 (7.22–36.15)	< 0.001*
Acne vulgaris	140 (22.5)	136 (25.2)	4 (4.9)	6.48 (2.33–18.04)	< 0.001*	6.47 (2.30–18.25)	< 0.001*
Rosacea	9 (1.4)	9 (1.7)	0	—	0.242	—	—
Psoriasis	2 (0.3)	2 (0.4)	0	—	0.583	—	—
Seborrheic dermatitis	40 (6.4)	40 (7.4)	0	—	0.011*	—	—
Xerosis	170 (27.4)	168 (31.1)	2 (2.5)	17.84 (4.33–73.44)	< 0.001*	18.32 (4.40–76.17)	< 0.001*
Urticaria	98 (15.8)	97 (18.0)	1 (1.2)	17.74 (2.44–129.02)	< 0.001*	17.43 (2.37–127.93)	0.005*
Dermatitis	106 (17.1)	104 (19.3)	2 (2.5)	9.42 (2.28–38.96)	0.002*	10.38 (2.48–43.57)	0.001*
History of atopic dermatitis, *n* (%)	163 (26.2)	158 (29.3)	5 (6.2)	6.30 (2.50–15.88)	< 0.001*	5.89 (2.32–14.98)	< 0.001*
History of family atopy, *n* (%)	273 (44.0)	242 (44.8)	31 (38.3)	1.31 (0.81–2.12)	0.270	—	—
Exercise, min/wk., median (IQR)	60.0 (20.0, 120.0)	60.0 (20.0, 120.0)	90.0 (30.0, 155.0)	1.00 (0.99–1.00)	0.128	—	—
Smoking, *n* (%)							
Current/past	40 (6.5)	32 (5.9)	8 (9.8)	0.58 (0.26–1.30)	0.182	—	—
Never	581 (93.6)	508 (94.1)	73 (90.1)	1.00 [ref]	1.00	—	—
Menstruation status, *n* (%)							
Reproductive age	338 (62.5)	356 (65.9)	32 (39.5)	1.59 (0.83–3.05)	0.163	—	—
Menopause	120 (19.3)	105 (19.4)	15 (18.5)	1.00 [ref]	1.00	—	—
Skin type, *n* (%)							
Normal skin	47 (7.6)	31 (5.7)	16 (19.8)	0.25 (0.13–0.45)	< 0.001*	0.28 (0.14–0.57)	< 0.001*
Dry skin	197 (31.7)	182 (33.7)	15 (18.5)	2.24 (1.254.01)	0.007*	2.18 (1.19–4.01)	0.012*
Oily skin	91 (14.7)	76 (14.1)	15 (18.5)	0.72 (0.39–1.33)	0.293	—	—
Mixed type	286 (46.1)	251 (46.5)	35 (43.2)	1.14 (0.71–1.83)	0.582	—	—
Fitzpatrick skin type, *n* (%)							
Type I–III	528 (85.0)	460 (85.2)	68 (84.0)	1.00 [ref]	1.000	—	—
Type IV–VI	93 (15.0)	80 (14.8)	13 (16.0)	0.91 (0.48–1.72)	0.772	—	—

Abbreviations: BMI, body mass index; CI, confidence interval; IQR, interquartile range; OR, odds ratio; SD, standard deviation. * *p* value ≤ 0.05 was considered statistical significance.

### 3.1 Sensitive Skin Severity Scores

In the sensitive skin group, the overall median (IQR) scores of the SS‐14, SS‐10, and BoSS questionnaires were 20 (7, 39.8), 17.5 (7–35), and 21 (13–31), respectively. In contrast, the nonsensitive skin group had median (IQR) scores of 2 (0–5) for the SS‐14, 1 (0–4.5) for the SS‐10, and 4 (0–14) for the BoSS. These scores were significantly lower than the corresponding values for individuals with sensitive skin (Table 2). According to sensitive skin severity, the SS‐14, SS‐10, and BoSS scores showed significant differences across mildly, moderately, and severely sensitive skin subgroups (*p* < 0.001; Table 2).

**TABLE 2:** Median scores of the SS‐14, SS‐10, and BoSS questionnaires for self‐reported sensitive skin severity.

Scores	Total participants (*n* = 621)	Sensitive skin (*n* = 540)	Nonsensitive skin (*n* = 81)	*p*
Median (IQR)				
SS‐14	17.0 (4.0, 36.5)	20.0 (7.0, 39.8)	2.0 (0.0, 5.0)	< 0.001*
SS‐10	14.0 (4.0, 31.0)	17.5 (7.0, 35.0)	1.0 (0.0, 4.5)	< 0.001*
BoSS	19.0 (10.0, 30.0)	21.0 (13.0, 31.0)	4.0 (0.0, 14.0)	< 0.001*

Scores	Mildly sensitive skin (*n* = 183)	Moderately sensitive skin (*n* = 250)	Severely sensitive skin (*n* = 107)	*p*
Median (IQR)				
SS‐14	7.0 (1.0, 17.0)	24.0 (13.8, 46.3)	39.0 (23.0, 76.0)	< 0.001*
SS‐10	7.0 (1.0, 14.0)	21.0 (12.0, 41.0)	34.0 (21.0, 67.0)	< 0.001*
BoSS	14.0 (7.0, 24.0)	22.0 (14.8, 31.3)	29.0 (20.0, 40.0)	< 0.001*

Abbreviations: BoSS, burden of sensitive skin; IQR, interquartile range; SS, sensitive scale.

* Independent‐samples Mann–Whitney *U* test: statistical significance set at a *p* value of < 0.05.

**FIGURE 1:**
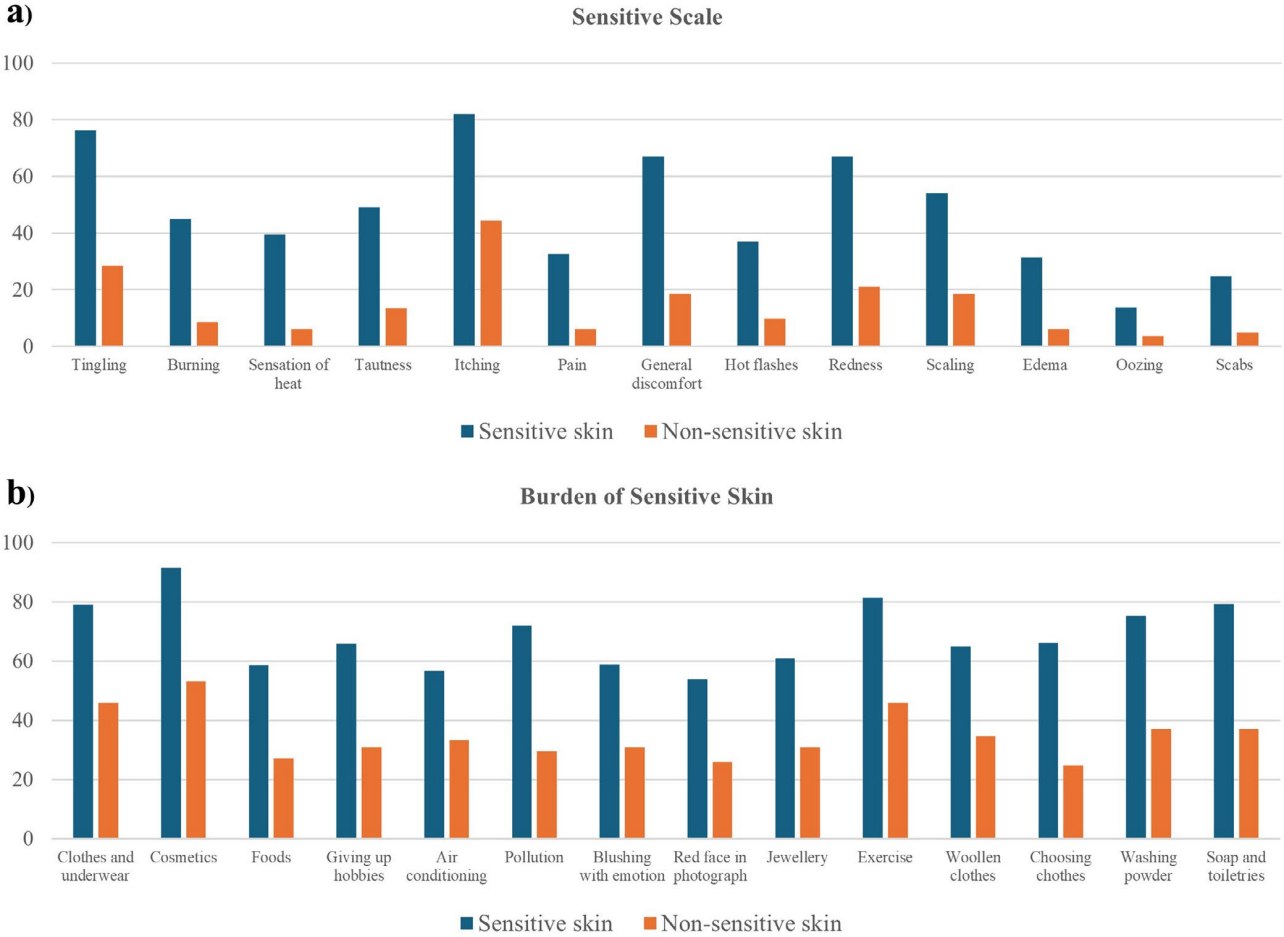
Percentage of reported sensitive skin symptoms and the impact on quality of life between sensitive and nonsensitive groups.

The most frequently reported symptom in the sensitive skin group was itching (82%), followed by tingling (76.3%) and general discomfort (66.9%). The most common clinical sign was redness (67.0%). In terms of the impact on quality of life, the BoSS questionnaire indicated that the most significant impact was difficulty in selecting and purchasing cosmetic products (91.5%), followed by flushing after exercise (81.5%) and being unable to use other people's soap and toiletries (79.3%; Figure 1).

### 3.2 Cutoff Scores for the Severity of Sensitive Skin

**FIGURE 2:**
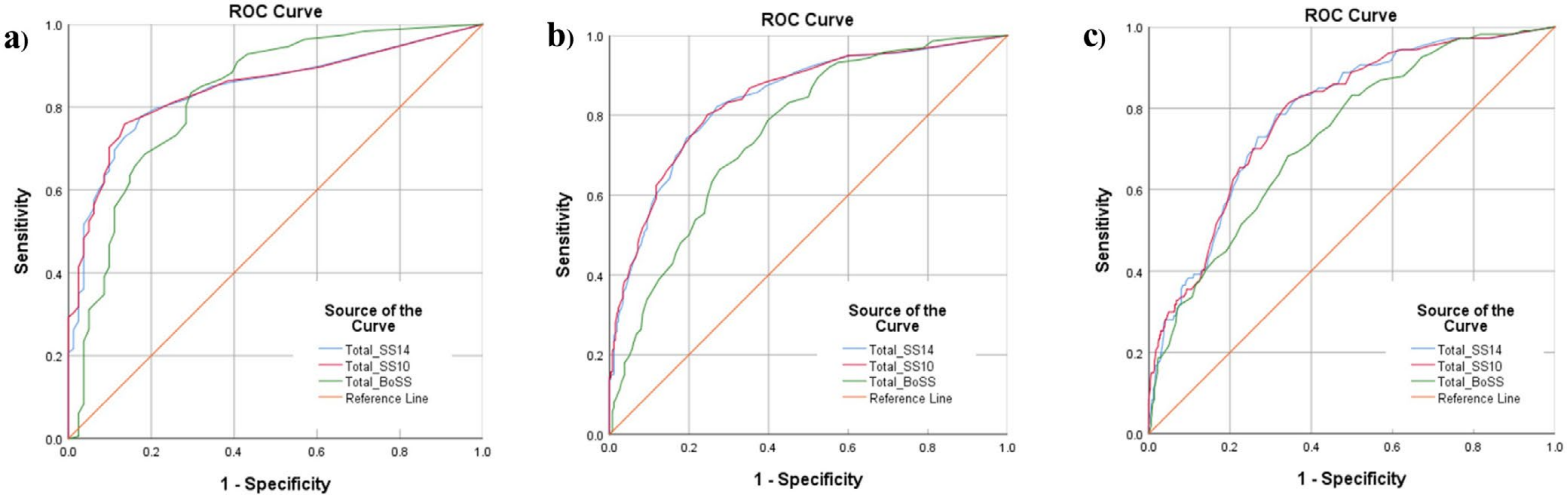
Comparison of area under the curve for the SS‐14, SS‐10, and BoSS questionnaires in (a) mildly sensitive, (b) moderately sensitive, and (c) severely sensitive skin subgroups.

This study established cutoff values for the SS‐14, SS‐10, and BoSS questionnaires to diagnose mildly, moderately, and severely sensitive skin. For mild disease, individuals who self‐reported having at least mild sensitive skin were considered positive cases for sensitive skin (*n* = 540; 86.9%). For moderate sensitive skin, patients self‐reporting at least moderate sensitive skin were considered positive cases (*n* = 357; 57.5%). Lastly, for severe sensitive skin, individuals self‐reporting severe sensitive skin were considered positive cases (*n* = 107; 17.23%). The ROC analysis yielded AUC values for these three severity levels (Table 3; Figure 2).

**TABLE 3:** Cutoff scores of the SS‐14, SS‐10, and BoSS questionnaires for diagnosing sensitive skin severity.

Scores	Severity	AUC ROC (95% CI)	Sensitivity (%)	Specificity (%)	Cutoff levels (≥)
SS‐14	Mild	0.842 (0.803–0.881)	79.6	79.0	6
	Moderate	0.839 (0.808–0.871)	77.9	76.1	16
	Severe	0.785 (0.739–0.830)	72.9	71.0	25
SS‐10	Mild	0.827 (0.772–0.881)	80.9	75.3	5
	Moderate	0.842 (0.881–0.873)	78.2	76.5	13
	Severe	0.786 (0.740–0.832)	72.0	71.0	22
BoSS	Mild	0.827 (0.772–0.881)	76.1	71.6	13
	Moderate	0.758 (0.719–0.796)	69.2	67.8	19
	Severe	0.730 (0.680–0.780)	68.2	65.8	24

Abbreviations: AUC, area under the curve; BoSS, burden of sensitive scale; CI, confidence interval; ROC, receiver operating characteristic; SS, sensitive scale.

The cutoff values for identifying sensitive skin were determined as follows: For mildly sensitive skin, the SS‐14 score was 6, the SS‐10 score was 5, and the BoSS score was 13. Regarding moderately sensitive skin, the SS‐14 score was 16, the SS‐10 score was 13, and the BoSS score was 19. As for severely sensitive skin, the SS‐14 score was 25, the SS‐10 score was 22, and the BoSS score was 24 (Table 3).

The AUC of the ROC curve for the SS‐10 questionnaire showed no significant differences compared with that of the SS‐14 questionnaire across all severity subgroups. However, both the SS‐14 and SS‐10 demonstrated significantly greater AUC values than the BoSS for the moderately sensitive subgroup. The SS‐10 also exhibited a significantly greater AUC than the BoSS for the severely sensitive subgroup (Figure 2; Table S1).

The calculated cutoff scores of 5, 13, and 22 on the SS‐10 were employed to diagnose mildly, moderately, and severely sensitive skin, respectively, among the participants. Based on these scores, the overall prevalence of sensitive skin was 73.6% (*n* = 457). Among these individuals, 25.4% (*n* = 116) were classified as having mildly sensitive skin, 25.2% (*n* = 115) as having moderately sensitive skin, and 49.4% (*n* = 226) as having severely sensitive skin.

### 3.3 Factors Influencing Sensitive Skin

The three most frequently reported aggravating factors for sensitive skin were hot weather (65.6%), air pollution (64.3%), and sunlight (55.7%). Other reported factors included cosmetic products (43.7%), dry air (38.9%), cold weather (31.9%), water (30.6%), humid air (20.4%), wind (18.1%), food (18%), emotional changes (17.8%), air conditioners (13.5%), medications (6.1%), and sweat (1.5%).

The potential risk factors associated with sensitive skin are detailed in Table 1. Sensitive skin was significantly more common in female patients (aOR 3.98, 95% CI 1.94–8.16; *p* < 0.001). The prevalence of underlying dermatologic conditions was greater in the sensitive skin group than in the nonsensitive skin group (aOR 16.15, 95% CI 7.22–36.15; *p* < 0.001). Specifically, the prevalence rates of acne vulgaris, xerosis, dermatitis, and urticaria were significantly greater in the sensitive skin group. Additionally, a history of atopic dermatitis was significantly more prevalent in the sensitive skin group (aOR 4.24, 95% CI 2.31–7.78; *p* < 0.001).

Regarding skin type, participants with dry skin had a significantly greater incidence of sensitive skin (aOR 2.18, 95% CI 1.19–4.01; *p* = 0.012), whereas those with normal skin were significantly less likely to have sensitive skin (aOR 0.28, 95% CI 0.14–0.57; *p* < 0.001). No statistically significant differences existed between the sensitive and nonsensitive skin groups in age, body mass index, Fitzpatrick skin type, physical exercise level, menstruation status, or smoking status.

Preexisting skin conditions and a history of atopic dermatitis were significantly more common in the severely sensitive skin subgroup than in the moderately and mildly sensitive subgroups (*p* < 0.001 and *p* = 0.01, respectively). No significant differences among the mildly, moderately, or severely sensitive skin subgroups were observed in age, body mass index, Fitzpatrick skin type, physical exercise level, menstruation status, or smoking status (Table S2).

## 4 Discussion

This study represents the first report on the prevalence, characteristics, and associated risk factors for sensitive skin in Thailand. Additionally, we determined the cutoff scores of the SS‐10, SS‐14, and BoSS questionnaires as diagnostic tools for sensitive skin and identified scoring levels for severity determination.

The prevalence of individuals who self‐reported sensitive skin at any level of severity was 86.9%, which is relatively high compared to findings from previous studies [4]. The high prevalence might be attributed to our self‐declaration questionnaire method, which was disseminated through public social platforms, potentially attracting more individuals with sensitive skin. However, when focusing specifically on self‐reported moderately or severely sensitive skin, the prevalence was 57.5%. A previous systematic review across 18 countries revealed that the global prevalence of self‐declared sensitive skin in the general population who claimed some degree of reactivity varied from 23% to 92%, with a pooled prevalence of 71% [4]. The overall prevalence of patients with moderately or very sensitive skin was 40% [4].

In Asian populations, prior studies from China, Japan, Korea, and India reported that the prevalence of moderately or severely sensitive skin ranged from 5% to 57%, with a pooled prevalence of 31% [4, 12, 13, 14, 15, 16]. The substantial variation in the prevalence of sensitive skin may be attributed to various factors, including different ethnicities, phototypes, languages, geographic regions, environmental variations, and survey methodologies across study populations.

Another key factor affecting the prevalence of sensitive skin is the diagnostic method used. The diagnosis of sensitive skin primarily relies on self‐report questionnaires, which are based on how patients subjectively perceive their skin's reactivity. However, the questionnaires used to diagnose sensitive skin vary, ranging from basic questions with or without definitions of sensitive skin to validated and more comprehensive questionnaires [5, 6, 17]. Consequently, the incidence of individuals identifying themselves as having sensitive skin without confirmation from dermatologists could be substantial. This situation may result from varying sensitivity thresholds among patients or misidentification because of underlying skin conditions or adverse treatment effects.

The SS questionnaires take a comprehensive approach, consisting of various subquestions about sensitive skin symptoms, which enhances the accuracy of the assessment compared to relying solely on patient self‐diagnosis. The SS‐10 questionnaire is preferred over the SS‐14 questionnaire because it focuses on sensitive symptoms and erythema, excluding other skin signs that are rarely observed in sensitive skin syndrome patients.

Previous studies have proposed different cutoff values for identifying sensitive skin using the SS questionnaire. For example, one study suggested that SS‐14 and SS‐10 scores exceeding 25 and 20, respectively, indicate sensitive skin [5]. Legeas et al. recently identified a more precise cutoff score of 12.7 for identifying French patients with very sensitive or sensitive skin [8], whereas Chan et al. established a threshold of 25.5 based on data from a Hong Kong population [7]. Interestingly, the cutoff scores for the SS‐10 questionnaire in our study were similar to those proposed by Legeas et al., where a score greater than 13 indicated moderately to severely sensitive skin, and individuals scoring less than 5 were categorized as not having sensitive skin.

Polena et al. suggested that the BoSS questionnaire could be a diagnostic tool for sensitive skin syndrome, with a score of at least 23 corresponding to very sensitive skin [10]. In our study, we found that a BoSS score of at least 24 indicated severely sensitive skin, whereas scores from 19 to 23.9 indicated moderately sensitive skin. Individuals scoring less than 13 points were categorized as not having sensitive skin. However, based on the DeLong test results, the SS questionnaire demonstrated superior performance in diagnosing sensitive skin compared to the BoSS questionnaire. Although the studies by Legeas et al. and Polena et al. were conducted on European populations, our findings revealed that these cutoff scores may also apply to Asian populations, including Thais. Interestingly, these cutoff values might establish universal benchmarks for a diagnostic tool for sensitive skin. Further study in other ethnic groups with darker Fitzpatrick skin types is recommended.

Our study identified several risk factors for sensitive skin, including female sex, underlying dermatologic conditions, a history of atopic dermatitis, and the dry skin subtype. The greater prevalence of sensitive skin in females may be attributed to several factors, including skin thickness, hormonal influences, cosmetic use, and underlying skin diseases. Females generally have thinner epidermis and dermis than males, making their skin more susceptible to irritation and reactivity [18].

Age is a known risk factor for sensitive skin, with studies suggesting that younger patients are more susceptible than older patients due to reduced cutaneous nerve innervation with age [19, 20]. Even though older individuals have reduced innervation, their increased use of anti‐aging cosmetics, which often contain acidic ingredients, such as retinol, retinal, hydroxyl acids, and ascorbic acid, may contribute to skin reactivity. Consequently, our study revealed no significant difference in the prevalence of sensitive skin across age groups.

Some studies have classified sensitive skin syndrome based on epidermal barrier function or the presence or absence of associated skin diseases [21, 22]. In primary sensitive skin, patients experience unpleasant symptoms without any underlying skin condition. In contrast, secondary sensitive skin refers to cases where patients have associated skin diseases, such as acne, rosacea, seborrheic dermatitis, and psoriasis [21, 23, 24]. Our study revealed that patients with sensitive skin have a greater incidence of underlying dermatologic diseases, including acne, xerosis, urticaria, and atopic dermatitis, than those without sensitive skin. Moreover, the severity of sensitive skin was linked to a higher proportion of participants with these underlying conditions. The impaired skin barrier function in certain dermatologic diseases could be a major contributing factor to increased skin reactivity. Furthermore, treatments or procedures used to manage dermatologic diseases, as well as cosmetic procedures, may cause skin irritation and increase the likelihood of developing sensitive skin. Skin type, especially dry skin, is another risk factor. The lack of skin lipids in dry skin increases skin permeability, affecting epidermal barrier function.

Environmental factors are also associated with sensitive skin. The equatorial location and tropical climate of Thailand result in hot weather and strong ultraviolet radiation, both of which are common aggravating factors for patients with sensitive skin. Exposure to ultraviolet B leads to epidermal barrier dysfunction and reduced hydration in the stratum corneum [25, 26]. Moreover, ultraviolet B radiation causes oxidative damage and inflammatory reactions in keratinocytes on the skin surface [27], thereby contributing to the development of sensitive skin. Exposure to high temperatures directly activates the transient receptor potential vanilloid (TRPV1) receptor, which plays a role in the pathophysiology of sensitive skin [28].

Pollution was the second most common aggravating factor in our study. In Thailand, fine particulate matter with a diameter less than 2.5 μm (PM2.5) is a significant environmental and public health concern. PM2.5 can increase skin permeability and trigger inflammatory responses, potentially leading to a greater risk of sensitive skin [29, 30]. Several studies have reported a significant association between air pollution and sensitive skin [15, 24]. However, direct evidence linking PM2.5 specifically to the development of sensitive skin remains lacking.

Our study had certain limitations. Since the data were self‐reported, the prevalence and some data might be overestimated or underestimated and should be interpreted cautiously, as with any questionnaire study. Furthermore, the generalizability of the data should be assessed, as the study might be based on a single population that may not represent the broader population.

## 5 Conclusions

Sensitive skin is a common condition in the Thai population. Factors associated with an increased risk include female sex, dry skin type, underlying dermatologic diseases, and a history of atopic dermatitis. Among the SS‐10, SS‐14, and BoSS questionnaires, the SS‐10 is the most suitable for diagnosing sensitive skin, with scores of 5, 13, and 22 corresponding to mildly, moderately, and severely sensitive skin, respectively. The determined cutoff values will assist in diagnosing sensitive skin and informing strategies for patient care.

## Author Contributions

W.B., S.K., P.C.: conception and design. P.C., S.P, S.L.: acquisition of data. S.P., S.L.: analysis and interpretation of data. P.C., N.V.: drafting the manuscript. P.C., S.K., W.B.: revising the manuscript for important intellectual content. All authors – final approval of the version to be published.

## Ethics Statement

This study was authorized by the Siriraj Institutional Review Board (approval number Si‐1021/2021) on December 20, 2021.

## Conflicts of Interest

The authors declare no conflicts of interest.

## Supporting information


Data S1.

## Data Availability

The datasets used and/or analyzed during the current study are available from the corresponding author upon reasonable request.

## References

[1] L. Misery , S. Ständer , J. C. Szepietowski , et al., “Definition of Sensitive Skin: An Expert Position Paper From the Special Interest Group on Sensitive Skin of the International Forum for the Study of Itch,” Acta Dermato‐Venereologica 97, no. 1 (2017): 4–6. 26939643 10.2340/00015555-2397

[2] A. Wollenberg and A. Giménez‐Arnau , “Sensitive Skin: A Relevant Syndrome, Be Aware,” Journal of the European Academy of Dermatology and Venereology 36, no. Suppl 5 (2022): 3–5. 10.1111/jdv.17903 35315153

[3] E. Berardesca , M. Farage , and H. Maibach , “Sensitive skin: an overview,” International Journal of Cosmetic Science 35, no. 1 (2013): 2–8. 22928591 10.1111/j.1468-2494.2012.00754.x

[4] W. Chen , R. Dai , and L. Li , “The Prevalence of Self‐Declared Sensitive Skin: A Systematic Review and Meta‐Analysis,” Journal of the European Academy of Dermatology and Venereology 34, no. 8 (2020): 1779–1788. 31869523 10.1111/jdv.16166

[5] L. Misery , C. Jean‐Decoster , S. Mery , V. Georgescu , and V. Sibaud , “A New Ten‐Item Questionnaire for Assessing Sensitive Skin: The Sensitive Scale‐10,” Acta Dermato‐Venereologica 94, no. 6 (2014): 635–639. 24710717 10.2340/00015555-1870

[6] L. Misery , E. Jourdan , S. Abadie , et al., “Development and Validation of a New Tool to Assess the Burden of Sensitive Skin (BoSS),” Journal of the European Academy of Dermatology and Venereology 32, no. 12 (2018): 2217–2223. 30022546 10.1111/jdv.15186

[7] K. T. M. Chan and A. H. N. Cheung , “Application of Receiver Operating Characteristic (ROC) Curve to Determine the Diagnostic Ability of A Validated Ten – Item Questionnaire (SS – 10) in Estimating the Prevalence of Sensitive Skin in Hong Kong Population,” International Journal of Innovative Research in Medical Science 4, no. 07 (2019): 405–413.

[8] C. Legeas , L. Misery , J. W. Fluhr , A. C. Roudot , A. S. Ficheux , and E. Brenaut , “Proposal for Cut‐Off Scores for Sensitive Skin on Sensitive Scale‐10 in a Group of Adult Women,” Acta Dermato‐Venereologica 101, no. 1 (2021): adv00373. 33426565 10.2340/00015555-3741 PMC9309873

[9] L. Misery , E. Jourdan , F. Huet , et al., “Sensitive Skin in France: A Study on Prevalence, Relationship With Age and Skin Type and Impact on Quality of Life,” Journal of the European Academy of Dermatology and Venereology 32, no. 5 (2018): 791–795. 29397030 10.1111/jdv.14837

[10] H. Polena , M. Chavagnac‐Bonneville , L. Misery , and M. Sayag , “Burden of Sensitive Skin (BoSS) Questionnaire and Current Perception Threshold: Use as Diagnostic Tools for Sensitive Skin Syndrome,” Acta Dermato‐Venereologica 101, no. 11 (2021): adv00606. 34648037 10.2340/actadv.v101.365 PMC9455310

[11] W. Boonchai , S. Kanokrungsee , S. Prasertsook , S. Likittanasombat , N. Viriyaskultorn , and P. Chaweekulrat , “Sensitive Skin in Thailand: Validity of Thai Versions of the Sensitive Scale and the Burden of Sensitive Skin Questionnaires,” Journal of Cosmetic Dermatology 23, no. 11 (2024): 3776–3778, 10.1111/jocd.16341. 39207019

[12] F. Xu , S. Yan , M. Wu , et al., “Self‐Declared Sensitive Skin in China: A Community‐Based Study in Three Top Metropolises,” Journal of the European Academy of Dermatology and Venereology 27, no. 3 (2013): 370–375. 22844976 10.1111/j.1468-3083.2012.04648.x

[13] X. Wang , Y. Su , B. Zheng , et al., “Gender‐Related Characterization of Sensitive Skin in Normal Young Chinese,” Journal of Cosmetic Dermatology 19, no. 5 (2020): 1137–1142. 31460701 10.1111/jocd.13123 PMC7047566

[14] Y. R. Kim , H. I. Cheon , L. Misery , C. Taieb , and Y. W. Lee , “Sensitive Skin in Korean Population: An Epidemiological Approach,” Skin Research and Technology 24, no. 2 (2018): 229–234. 29067709 10.1111/srt.12418

[15] R. Kamide , L. Misery , N. Perez‐Cullell , V. Sibaud , and C. Taïeb , “Sensitive Skin Evaluation in the Japanese Population,” Journal of Dermatology 40, no. 3 (2013): 177–181. 23253054 10.1111/1346-8138.12027

[16] E. Brenaut , L. Misery , and C. Taieb , “Sensitive Skin in the Indian Population: An Epidemiological Approach,” Front Med (Lausanne) 6 (2019): 29. 30842946 10.3389/fmed.2019.00029 PMC6391320

[17] M. Corazza , F. Guarneri , L. Montesi , G. Toni , I. Donelli , and A. Borghi , “Proposal of a Self‐Assessment Questionnaire for the Diagnosis of Sensitive Skin,” Journal of Cosmetic Dermatology 21, no. 6 (2022): 2488–2496. 34553479 10.1111/jocd.14425 PMC9292491

[18] S. Rahrovan , F. Fanian , P. Mehryan , P. Humbert , and A. Firooz , “Male Versus Female Skin: What Dermatologists and Cosmeticians Should Know,” International Journal of Women's Dermatology 4, no. 3 (2018): 122–130. 10.1016/j.ijwd.2018.03.002 PMC6116811 30175213

[19] A. F. de Bengy , J. Lamartine , D. Sigaudo‐Roussel , and B. Fromy , “Newborn and Elderly Skin: Two Fragile Skins at Higher Risk of Pressure Injury,” Biological Reviews of the Cambridge Philosophical Society 97, no. 3 (2022): 874–895. 34913582 10.1111/brv.12827

[20] X. Chen , J. Wen , W. Wu , Q. Peng , X. Cui , and L. He , “A Review of Factors Influencing Sensitive Skin: An Emphasis on Built Environment Characteristics,” Frontiers in Public Health 11 (2023): 1269314. 38111482 10.3389/fpubh.2023.1269314 PMC10726041

[21] A. Guerra‐Tapia , E. Serra‐Baldrich , L. Prieto Cabezas , E. González‐Guerra , and J. L. López‐Estebaranz , “Diagnosis and Treatment of Sensitive Skin Syndrome: An Algorithm for Clinical Practice,” Actas Dermo‐Sifiliográficas (English Edition) 110, no. 10 (2019): 800–808. 10.1016/j.ad.2018.10.021 31146882

[22] T. Yokota , M. Matsumoto , and T. Sakamaki , “Classification of Sensitive Skin and Development of a Treatment System Appropriate for Each Group,” IFSCC Magazine 6 (2003): 303–307.

[23] L. Misery , S. Boussetta , T. Nocera , N. Perez‐Cullell , and C. Taieb , “Sensitive skin in Europe,” Journal of the European Academy of Dermatology and Venereology 23, no. 4 (2009): 376–381. 19335729 10.1111/j.1468-3083.2008.03037.x

[24] L. Misery , V. Sibaud , C. Merial‐Kieny , and C. Taieb , “Sensitive Skin in the American Population: Prevalence, Clinical Data, and Role of the Dermatologist,” International Journal of Dermatology 50, no. 8 (2011): 961–967. 21781068 10.1111/j.1365-4632.2011.04884.x

[25] S. J. Jiang , A. W. Chu , Z. F. Lu , M. H. Pan , D. F. Che , and X. J. Zhou , “Ultraviolet B‐Induced Alterations of the Skin Barrier and Epidermal Calcium Gradient,” Experimental Dermatology 16, no. 12 (2007): 985–992. 18031457 10.1111/j.1600-0625.2007.00619.x

[26] Z. Liu , S. Song , W. Luo , P. M. Elias , and M. Q. Man , “Sun‐Induced Changes of Stratum Corneum Hydration Vary With Age and Gender in a Normal Chinese Population,” Skin Research and Technology 18, no. 1 (2012): 22–28. 21507068 10.1111/j.1600-0846.2011.00536.x

[27] T. A. Lee , Y. T. Huang , P. F. Hsiao , L. Y. Chiu , S. R. Chern , and N. L. Wu , “Critical Roles of Irradiance in the Regulation of UVB‐Induced Inflammasome Activation and Skin Inflammation in Human Skin Keratinocytes,” Journal of Photochemistry and Photobiology. B 226 (2022): 112373. 10.1016/j.jphotobiol.2021.112373 34959183

[28] M. J. Caterina , M. A. Schumacher , M. Tominaga , T. A. Rosen , J. D. Levine , and D. Julius , “The Capsaicin Receptor: A Heat‐Activated Ion Channel in the Pain Pathway,” Nature 389, no. 6653 (1997): 816–824. 9349813 10.1038/39807

[29] B. E. Kim , J. Kim , E. Goleva , et al., “Particulate Matter Causes Skin Barrier Dysfunction,” JCI Insight 6, no. 5 (2021): e145185, 10.1172/jci.insight.145185. 33497363 PMC8021104

[30] C. Le Gall‐Lanotto , A. Verdin , F. Cazier , et al., “Road‐Traffic‐Related Air Pollution Contributes to Skin Barrier Alteration and Growth Defect of Sensory Neurons,” Experimental Dermatology 33, no. 1 (2024): e15009. 38284185 10.1111/exd.15009

