# Supplementary material for: Sensitive Skin in Thais: Prevalence, Clinical Characteristics, and Diagnostic Cutoff Scores
Source: J Cosmet Dermatol. 2025 Apr 11;24(4):e70181. doi: 10.1111/jocd.70181 (PMC11986799; doi:10.1111/jocd.70181)
Supplement: Supplementary file 1 — Data S1. [file JOCD-24-e70181-s001.docx]

**Supplementary Table 1** Comparison of the area under the curve among sensitive skin questionnaires

| **Mildly sensitive skin** | | | |
| --- | --- | --- | --- |
| **Scores** | **Area under curve** | **SS-14** | **SS-10** |
| **SS-14** | 0.842 |  |  |
| **SS-10** | 0.845 | 0.375 |  |
| **BoSS** | 0.827 | 0.644 | 0.556 |
| **Moderately sensitive skin** | | | |
| **Scores** | **Area under curve** | **SS-14** | **SS-10** |
| **SS-14** | 0.839 |  |  |
| **SS-10** | 0.842 | 0.251 |  |
| **BoSS** | 0.758 | **<0.001*** | **<0.001*** |
| **Severely sensitive skin** | | | |
| **Scores** | **Area under curve** | **SS-14** | **SS-10** |
| **SS-14** | 0.785 |  |  |
| **SS-10** | 0.786 | 0.764 |  |
| **BoSS** | 0.730 | 0.056 | **0.048*** |

**Abbreviations:** BoSS, Burden of Sensitive Skin; DLQI, Dermatology Life Quality Index; SS, Sensitive Scale

**Supplementary Table 2** Factors influencing sensitive skin among mildly, moderately, and severely sensitive skin subgroups

|  | **Mildly sensitive skin**  (n=183) | **Moderately sensitive skin** (n=250) | **Severely sensitive skin**  (n=107) | ***P* value** |
| --- | --- | --- | --- | --- |
| **Sex**, n (%) |  |  |  |  |
| Female | 154 (84.2) | 211 (84.4) | 95 (88.8) | 0.503 |
| Male | 29 (15.8) | 39 (15.6) | 12 (11.2) |  |
| **Age** y, mean±SD | 40.03 ± 14.25 | 39.17 ± 12.03 | 39.55 ± 38.0 | 0.857 |
| **BMI,** kg/m^2^, median (IQR) | 22.3 (20.0,24.8) | 22.7 (20.1,26.4) | 22.8 (20.2,27.1) | 0.149 |
| **Underlying dermatologic disease**,  n (%) | 72 (39.3) | 170 (68.0) | 84 (78.5) | **<0.001*** |
| **History of atopic dermatitis**, n (%) | 69 (37.7) | 126 (50.4) | 57 (53.3) | **0.010*** |
| **History of family atopy**, n (%) | 84 (45.9) | 103 (41.2) | 55 (51.2) | 0.193 |
| **Exercise**, min/wk,  median (IQR) | 60.0 (30.0,150.0) | 60.0 (20.0,120.0) | 60.0 (20.0,120.0) | 0.167 |
| **Smoking**, n (%) |  |  |  |  |
| Current/past | 10 (5.5) | 16 (6.4) | 6 (5.6) | 0.909 |
| Never | 173 (94.5) | 234 (93.6) | 101 (94.4) |  |
| **Menstruation status**, n (%) |  |  |  |  |
| Reproductive age | 113 (61.7) | 168 (67.2) | 75 (70.1) | 0.362 |
| Menopause | 41 (22.4) | 43 (17.2) | 21 (19.6) |  |
| **Skin type**, n (%) |  |  |  |  |
| Normal skin | 15 (8.2) | 15 (6.0) | 1 (0.9) | **0.036** |
| Dry skin | 64 (35.0) | 71 (28.4) | 47 (43.9) | **0.016** |
| Oily skin | 20 (10.9) | 42 (16.8) | 14 (13.1) | 0.210 |
| Mixed type | 84 (45.9) | 122 (48.8) | 45 (42.1) | 0.495 |
| **Fitzpatrick skin type**, n (%) |  |  |  |  |
| Type I-III | 160 (87.4) | 206 (82.4) | 94 (87.9) | 0.238 |
| Type IV-VI | 23 (12.6) | 44 (17.6) | 13 (12.1) |  |

**Abbreviations:** BMI, body mass index; IQR, interquartile range
